# Exome-based cancer predisposition gene testing can provide a genetic diagnosis for individuals with heterogeneous tumor phenotypes

**DOI:** 10.1038/s41431-025-01814-z

**Published:** 2025-02-20

**Authors:** Snežana Hinić, Arjen R. Mensenkamp, Janneke H. M. Schuurs-Hoeijmakers, Fulvia Brugnoletti, Lilian Vreede, Elke M. van Veen, Barend Mijzen, Rachel S. van der Post, Maurizio Genuardi, Marjolijn J. L. Ligtenberg, Nicoline Hoogerbrugge, Richarda M. de Voer

**Affiliations:** 1 Department of Human Genetics, Research Institute for Medical Innovation, Radboud university medical center, Nijmegen, Netherlands; 2 Genomic Medicine, Department of Life Sciences and Public Health, Università Cattolica del Sacro Cuore, Rome, Italy; 3 European Reference Network for Genetic Tumour Risk Syndromes (ERN GENTURIS), Nijmegen, Netherlands; 4 Department of Pathology, Research Institute for Medical Innovation, Radboud university medical center, Nijmegen, Netherlands; 5 Medical Genetics Unit, Fondazione Policlinico Universitario A. Gemelli IRCCS, Rome, Italy

**Keywords:** Cancer genomics, Cancer genetics, Genetics research

## Abstract

The development of multiple primary tumors is one of the hallmarks of hereditary cancer. The phenotypic presentation of individuals with multiple primary tumors is often heterogeneous, which hampers the establishment of a genetic diagnosis. The absence of a genetic diagnosis may lead to inappropriate surveillance advices and treatment choices. The aim of this study was to investigate whether whole-exome sequencing (WES) and variant prioritization in all genes associated with cancer predisposition can identify pathogenic variants that explain the phenotypes of individuals who developed multiple primary tumors. Here, we report the findings of exome-based cancer predisposition gene testing in individuals (*n* = 72) who presented with multiple primary tumors (both malignant and benign) before the age of 65 years. Overall, a germline pathogenic variant (gPV) in a cancer predisposing gene was identified in 9.7% of individuals (*CHEK2*, *FANCM*, *NF1*, *POT1* and *PTEN*) and a candidate variant in 4.2% of individuals (*HOXB13*, *MAX* and *RECQL4*). Furthermore, by analyzing variants that occur in genes in cancer-associated pathways, we identified a candidate gene (*RECQL5*) for further follow-up. In conclusion, our study indicates that exome-based cancer predisposition gene testing may aid in the identification of pathogenic variants in individuals who developed multiple primary tumors. Our findings demonstrate that individuals with gPVs in genes associated with cancer predisposition may present with a broad tumor spectrum.

## Introduction

Hereditary cancer is rare and accounts for 5–10% of all cancers [1]. The main indications for hereditary cancer (‘hereditary cancer hallmarks’) include an early-age of tumor onset, familial aggregation of cancer types and/or the development of multiple primary tumors, both malignant and benign, in a single individual [2]. Besides inherited germline pathogenic variants (gPVs), multiple primary tumors can develop due to other factors, such as exposure to carcinogenic substances, like cigarette smoke, UV-light and asbestos [3]. However, therapeutic interventions, such as radiotherapy and/or chemotherapies to treat a prior malignancy may also underlie the development of multiple primary tumors [4]. The clinical presentation of individuals who develop multiple primary tumors is heterogeneous. The combination of different malignant tumor types may not fit with a well-known genetic tumor risk syndrome. Furthermore, rare genetic tumor risk syndromes, such as *PTEN* hamartoma tumor syndrome or Neurofibromatosis, can have heterogeneous phenotypic presentations [5]. This phenotypic heterogeneity may hamper the establishment of a genetic diagnosis that is instrumental for genetic counseling, surveillance advices for patients and their relatives, and treatment choices.

Current clinical and diagnostic practices rely on recognizing hereditary cancer hallmarks and offering genetic testing to individuals suspected for hereditary cancer. Gene panel testing in line with the observed phenotype is usually standard-of-care and more affordable and faster in comparison to whole-exome sequencing (WES) in most genetic diagnostic centers [6]. For example, testing only for genes involved in polyposis and colorectal cancer is usually preformed in individuals who have developed multiple adenomatous polyps. The main downside of gene panel testing is that the causative gPV can be missed when there is not a clear indication of which genes to test, which is often the case in individuals who developed multiple primary tumors. For these individuals, a more suitable option can be testing for a large gene panel that includes well-known cancer susceptibility genes or performing WES where the whole exome, including known and candidate cancer genes, will be analyzed. The latter approach has proven successful in identifying causative gPVs, in establishing potential novel genotype-phenotype associations for other rare genetic diseases [7, 8], and allows for re-analyses when novel genes for cancer susceptibility have been identified.

Here, we investigated the yield of germline pathogenic variants from exome-based cancer predisposition gene testing (*n* = 317 genes) in individuals (*n* = 72) who developed two or more tumors. Furthermore, we investigated whether any likely g(L)PVs occurred in genes in cancer-associated pathways.

## Materials and methods

### Study cohort

The retrospective study cohort consists of 72 individuals who presented with two or more tumors of which at least one tumor was malignant and developed before the age of 65 years. Individuals included in the cohort were genetically counselled at the Department of Human Genetics of Radboudumc in Nijmegen, the Netherlands (*n* = 55) or the Genetics Department of Fondazione Policlinico Gemelli in Rome, Italy (*n* = 17). We retrieved all cases who developed tumors and underwent whole-exome sequencing (WES), -analysis and interpretation in diagnostics between 2017 and 2022 at Radboudumc in Nijmegen, the Netherlands. Subsequently, we selected cases that fulfilled our criteria of having developed two or more tumors of which at least one tumor was malignant and developed before the age of 65 years and provided written informed consent for research. In total, 35 cases fulfilled these criteria and are further referred to as “diagnostic cohort” throughout the manuscript. All cases, except one, underwent WES in the diagnostic setting after targeted gene (panel) testing (e.g. Sanger- or next-generation sequencing of a single or small set of gene(s)) did not or likely not explain the full phenotypic spectrum of the respective individual. One individual was directly whole-exome sequenced, without targeted gene (panel) testing. In parallel, 37 cases who developed multiple primary tumors who were also without a genetic diagnosis after negative targeted gene (panel) testing were selected from the biobank Genetics and Rare Diseases at Radboudumc (*n* = 20) and from the local research database from Rome (*n* = 17), and subjected to WES (further referred to as “research cohort” throughout the manuscript). The phenotype of each case included in this study is outlined in Supplementary Table 1. All individuals have provided informed consent and the study was approved by the local ethics committee of the Radboudumc (study number 2019–5738) and Fondazione Policlinico Gemelli (study number 3577).

### Whole exome sequencing of germline DNA from blood

In brief, genomic DNA was extracted from peripheral blood and WES libraries were prepared using the Agilent SureSelectXT Human All Exon v4 (*n* = 6) or v5 (*n* = 66; Agilent Technologies, Santa Clara, CA) enrichment kits according to the manufacturer’s instructions. Next, libraries were sequenced at 2x150bp paired-end reads, and obtained reads were demultiplexed and aligned to the reference genome (build GRCh37) using Burrows-Wheeler Aligner (BWA). Subsequently, small indels and single nucleotide variants (SNVs) were called using Genome Analysis Toolkit (GATK) HaplotypeCaller (Broad Institute, following best practices) and annotated using an in-house developed annotation pipeline described previously [9].

### Germline variant prioritization

After sequencing and collection of the annotated variant files, all exomes underwent germline variant prioritization. In brief, all variants covered by ≥15 sequencing reads and ≥5 variant reads were selected. Subsequently, we prioritized all: i) (predicted) loss-of-function (LOF) variants (nonsense, frameshift and canonical splice site); ii) missense and in-frame indel variants with a Phylop score ≥3 and CADD_Phred score ≥15; and iii) synonymous variants and canonical splice site variants with a SpliceAI score of >0.2. Next, the variants were split into two groups: i) variants in genes associated with cancer predisposition (*n* = 317; Supplementary Table 2) and ii) variants in genes involved in the following cancer-associated pathways: replication and repair (KEGG 2.4), cell growth and cell death (KEGG 4.2), cancer (KEGG 6.1 and 6.2) and signal transduction (KEGG 3.2) or variants in genes that were annotated with the Gene ontology (GO) term “DNA repair” (Supplementary Table 3). To extract the list of genes associated with the abovementioned pathways, limma and org.Hs.eg.db packages were used in R version 4.1 via RStudio.

For genes associated with cancer predisposition, we prioritized variants further for autosomal-recessive (AR) and autosomal-dominant (AD) modes of inheritance. For variants potentially fulfilling the AR mode of inheritance, only variants with a minor allele frequency (MAF) ≤ 0.5 in our in-house database of >25.000 exomes, gnomAD and ExAC databases were kept. For variants that may act in an AD mode of inheritance, only variants with MAF ≤ 0.05 in the aforementioned databases were kept (Supplementary Fig. 1).

For variants in genes involved in cancer-associated pathways, we kept all variants potentially inherited in an AR way as described above. Variants in these genes that were considered in the AD mode of inheritance were only investigated when a MAF of ≤0.002 in our in-house and gnomAD databases was observed and if they occurred ≤2 times in ExAC. All prioritized variants were interpreted according to the American College of Medical Genetics and Genomics and the Association for Molecular Pathology (ACMG/AMP) criteria. Germline (likely) pathogenic variants identified in exomes that were sequenced in the diagnostic setting were cross-checked if they were indeed previously identified. Variants (e.g. (likely) pathogenic or variants of unknown significance) described in this manuscript (Table 1) are submitted to the Leiden Open Variant Database (LOVD; https://databases.lovd.nl/).

**Table 1:** Overview of germline (likely) pathogenic variants and variants of unknown significance identified by exome-based cancer predisposition gene testing of individuals who developed multiple primary tumors.

Gene	Patient ID	Sex (F/M)	(Pre)malignancies (age)^a^	Benign lesions and other clinical features (age)^a^	Transcript ID	Genomic position (genome build hg19)	cDNA position	Protein change	Zygosity	SpliceAI score	ACMG classification	ACMG pathogenicity	Diagnostic analyses performed^b^	Cohort^c^	Study
*CHEK2*	UPN001	M	Dysplastic nevi skin (40); Mel (43); TC (49); Essential thrombocythemia (59); CRC (60); CLL (64); Bowen’s disease (66); PC (67); Skin (67)	Hemangioma skin (37); Nevi naevocellularis (37, 38, 44); BCC (43, 51, 66)*; Lipoma subcutis (53); Pol (64)	NM_007194.4	chr22: g.29091857del	c.1100del	p.(Thr367MetfsTer15)	hom	NA	PVS1; PM2; PS4	Pathogenic	*MSH2*, *MSH6*, WES panel hereditary cancer (DG-2.5)**	D	ref15^#^
*CHEK2*	UPN008	F	TC (53); EC (53); CIS-AD (54); BC (54); UCC (62)	Pol (53, 54, 55, 57, 64, 64); Nevi naevocellularis (54)	NM_007194.4	chr22: g.29091857del	c.1100del	p.(Thr367MetfsTer15)	hom	NA	PVS1; PM2; PS4	Pathogenic	*PTEN*, WES panel hereditary cancer (DG-2.13)**	D	ref15^#^
*CHEK2*	UPN009	M	CRC (64); CRC (64)	Lipoma (63); Trichilemmoma (39); Pol (64)	NM_007194.4	chr22: g.29091857del	c.1100del	p.(Thr367MetfsTer15)	hom	NA	PVS1; PM2; PS4	Pathogenic	*PTEN*	R	ref15^#^
*FANCM*	UPN102	F	HnNC (40); OrC (40); EsC (49)	NR	NM_020937.4	chr14: g.45636336 C > T	c.1972C>T	p.(Arg658Ter)	hom	NA	PVS1; PM3; PM2; PS3	Pathogenic	WES panel hereditary cancer (DG-2.12)**	D	This study
*NF1*	UPN126	F	TC (48); DCIS (51); BC (51)	Thyroid nodules; Breast fibroadenomas; Multiple neurofibromas; Cafe-au lait spots	NM_001042492.3	chr17: g.29553697 C > G	c.2246 C > G	p.(Ser749Ter)	het	NA	PVS1; PM2; PS4	Pathogenic	*PTEN*, *BRCA1*, BRCA2, *CHEK2* c.1100del	R	This study
*POT1*	UPN232	F	OvC (43); Mel (55, 72, 74); TC (60); Ly (60); LC (62); Sarcoma (79)	BCC (60)*	NM_015450.3	chr7: g.124510964 C > A	c.255+1 G > T	p.?	het	0.9762	PVS1; PM2; PS4	Pathogenic	*BRCA1*, *BRCA2*, WES panel hereditary cancer (DG-2.18)**	D	This study
*PTEN*	UPN133	F	BC (45); EC (46)	Atypical breast fibroadenoma (33); Bra (46); Pancreas tumor (47)	NM_000314.8	chr10: g.89690851 G > C	c.253+5 G > C	p.?	het	0.848	PP3; PM2; PS1	Likely pathogenic	*PTEN*^$^, *TP53*, *BRCA1*, *BRCA2*	R	This study
*HOXB13*	UPN090	M	Mel (63); PC (64); LC (70)	NR	NM_006361.6	chr17: g.46805705 C > T	c.251 G > A	p.(Gly84Glu)	het	NA	PM2; PP1	Pathogenic	WES panel hereditary cancer (DG_2.4.1)**	D	This study
*MAX*	UPN078	M	PGC (31)	Adr (51); Acromegaly	NM_002382.5	chr14: g.65544706 T > C	c.220 A > G	p.(Met74Val)	het	NA	PM2; PP3; PM1; PP2	VUS	*MAX*, *MEN1*, WES panel hereditary cancer (DG-2.10)**	D	This study
*RECQL4*	UPN114	M	BlC (49); CRC (63)	Pol (63); Macrocephaly	NM_004260.4	chr8: g.145737770 C > T	c.3055+5 G > A	p.?	comp het	0.4039	PM2; PP3	VUS	*APC*, *MUTYH*, *PTEN*	R	This study
						chr8: g.145739073 G > A	c.2082 C > T	p.(Gly694 = )		0.5161	PM2; PP3	VUS			

*ACMG* American College of Medical Genetics and Genomics, *Adr* adrenal gland tumor, *BC* breast cancer, *BCC* basal cell carcinoma, *BlC* bladder cancer, *Bra* brain tumor, *cDNA* complementary DNA, *CIS-AD* carcinoma in situ-adenoma, *CLL* chronic lymphocytic leukemia, *com het* compound heterozygous, *CRC* colorectal cancer, *D* diagnostics, *DCIS* ductal carcinoma in situ, *DG* version of a gene panel (internal Radboudumc label), *EC* endometrial cancer, *EsC* esophageal carcinoma, *F* female, *het* heterozygous, *hg19* human genome build 19, *HnN* head and neck tumor, *hom* homozygous, *LC* lung cancer, *Ly* lymphoma, *M* male, *Mel* melanoma, *NR* not reported, *OrC* oropharyngeal carcinoma, *OvC* ovarian carcinoma, *PC* prostate cancer, *PGC* pituitary gland carcinoma, *Pol* colorectal polyp, *R* research, *TC* thyroid cancer, *UCC* urothelial carcinoma, *VUS* variant of uncertain significance, *WES* whole-exome sequencing.

*Counted as benign due to its benign behavior.

**WES panel hereditary cancer (DG_version) includes panel of genes associated with cancer development. This panel is regularly updated at Radboudumc. For specific versions of the gene panel see: https://www.radboudumc.nl/en/patient-care/patient-examinations/exome-sequencing-diagnostics/exomepanelspreviousversions/exomepanelspreviousversions/hereditary-cancer.

^#^Cases and their UPN IDs are identical to Hinić et al., (2024).

^$^The PTEN variant was previously during targeted gene testing not considered pathogenic at that time.

^a^Number in the brackets denotes the age at which a tumor/cancer was diagnosed.

^b^Reports all previous analyses in the diagnostic setting for the respective individual.

^c^Variant was identified though WES in the diagnostic cohort (D) or research cohort (R).

### In-silico variant effect prediction tools

To predict the effect of a missense variant on protein function, we used the online application MetaDome [10]. To visualize the location of the variant in the 3D structure of the protein, we used the SWISS-MODEL online application [11] and the protein structure was predicted by AlphaFold [12]. Last, to investigate the structural effects of a missense variant in the protein sequence, we used the online tool HOPE [13].

### Statistical analysis

To investigate the age of onset differences between males and females, an unpaired *t*-test was performed. *P* values <0.05 were considered statistically significant. All statistical analyses were performed in GraphPad Prism (version 9.5.0).

## Results

### Clinical characteristics of the study cohort

**Fig. 1:**
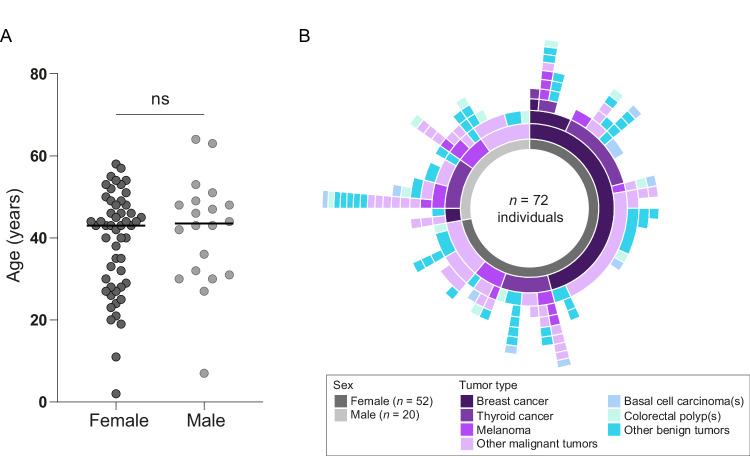
Cohort characteristics of individuals who developed multiple primary tumors. **A** Age at first malignant tumor diagnosis for females and males. **B** Tumor phenotypes (both malignant and benign) are shown in different colors. Three most common malignancies are shown separately, and all other malignancies are grouped as “other malignant”. When colorectal polyps or basal cell carcinomas were observed, they were plotted only once, as it is not uncommon to develop multiple colorectal polyps and basal cell carcinomas. More details on full phenotypes can be found in Supplementary Table 1.

In total, seventy-two individuals who developed multiple primary tumors were included in this descriptive study. The majority of individuals were female (*n* = 52; 72.2%; Supplementary Table 1). The median age at first malignant tumor diagnosis was 43 years [range 2–64] (Fig. 1A), which is not significantly different between females (43 years; [range 2–58]) and males (43.5 years; [range 7–64]; *P* = .41). The median number of tumors per individual was 3 [range 2–18]. Malignant tumors in the cohort were of 22 different origins and the most common malignancy types were breast cancer (*n* = 40; 35 individuals), thyroid cancer (*n* = 27; 27 individuals) and melanoma (*n* = 25; 17 individuals; Fig. 1B).

### (Likely) pathogenic variants and variants of unknown significance in genes associated with cancer predisposition

In total, in the exomes of 10 individuals (10/72; 13.9%) g(L)PVs (8 individuals) and variants of unknown significance (VUS; 2 individuals) in genes associated with cancer predisposition were identified. Six of these individuals were genetically diagnosed through diagnostic WES analysis (6/35; 17.1%; Table 1**;** Supplementary Table 1) and four through WES analysis in the research cohort (4/37; 10.8%; Table 1**;** Supplementary Table 1). Below, we describe our findings in further detail.

In three individuals (two males; UPN001 and UPN009, and a female; UPN008), a homozygous frameshift variant in *CHEK2* (NM_007194.4; c.1100delC; p.(Thr367MetfsTer15)) was identified (Table 1**;** Supplementary Table 1). This gPV is a founder variant and the most common gPV in *CHEK2* in West European populations [14]. The full phenotypic presentation of these individuals is described in Hinić et al. (2024) where we describe that individuals with biallelic gPVs in *CHEK2* may be susceptible to develop multiple malignancies in various tissues [15].

Another homozygous variant was identified in a female (UPN102). She presented with a homozygous nonsense variant in *FANCM* (NM_020937.4; c.1972C > T; p.(Arg658Ter)) that was located in a region of homozygosity that was approximately 32 Mb in size (Supplementary Fig. 2A). UPN102 developed a head and neck (jaw) tumor and an oropharyngeal cancer at the age of 40 years, as well as an esophageal cancer at the age of 49 years (Table 1). Individuals with biallelic *FANCM* gPVs have been observed to develop several types of cancer including breast- and squamous cell carcinoma at a young age [16, 17]. Furthermore, *FANCM* (OMIM: 609644) is linked to autosomal-recessive premature ovarian failure and spermatogenic failure.

Another female (UPN126) presented with a heterozygous nonsense variant in *NF1* (NM_ 001042492.3; c.2246 C > G; p.(Ser749Ter)). UPN126 was diagnosed with a papillary thyroid cancer at the age of 48 years, two breast (pre-)malignancies at the age of 51 years and benign thyroid nodules, breast fibroadenomas, multiple neurofibromas and café-au-lait spots (Table 1). Germline PVs in *NF1* lead to neurofibromatosis type I, a disorder characterized by fibromatous tumors and café-au-lait spots and individuals with this disorder have an increased susceptibility to develop malignant and benign tumors (OMIM: 162200).

A gPV in *PTEN* (NM_000314.8; c.253+5 G > C) with a predicted splice donor site loss was identified in a female (UPN133) who developed an atypical breast fibroadenoma at the age of 33 years, a breast cancer at the age of 45 years, and an endometrial cancer and a benign pancreatic tumor and a meningioma at the age of 46 years (Table 1). Germline PVs in *PTEN* cause a PTEN-hamartoma tumor syndrome, a phenotypically heterogeneous genetic disorder that includes benign hamartomatous tumors and malignant tumors including breast-, thyroid-, renal-, endometrial-, colorectal cancer and melanoma [5]. Other gPVs located on the same position (c.253+5) in *PTEN* are described to be pathogenic [18]. A mini-gene assay, in which this variant was tested, indicates that this variant causes aberrant splicing (data not shown).

A predicted splice donor site loss variant was identified in *POT1* (NM_015450.3; c.255+1 G > T) in a female (UPN232; Table 1) who presented with an ovarian cancer at the age of 43 years, multiple melanomas (ages 55, 72 and 74 years), a thyroid cancer, a lymphoma and a basal cell carcinoma (all at the age of 60 years), a lung cancer at the age of 62 years and a soft tissue sarcoma at the age of 79 years (Table 1). *POT1* is involved in telomere maintenance and gPVs in *POT1* predispose to development of multiple malignant and benign tumors (OMIM: 615848). RNA analysis performed in diagnostics confirmed that this variant affects *POT1* splicing (data not shown), and as such this variant is classified as pathogenic. Furthermore, the length of the telomeres was elongated in lymphocytes of this individual ( >99^th^ percentile).

As mentioned above, in three individuals, we identified a gPV and VUSs that do not completely explain the tumor phenotype of the respective individual. A heterozygous missense gPV in *HOXB13* (NM_006361.6; c.251 G > A; p.(Gly84Glu)) was identified in a male (UPN090) with a melanoma at the age of 63 years, a prostate cancer at the age of 64 years and a lung cancer at the age of 70 years (Table 1). Germline variants in *HOXB13* are known risk factors for prostate cancer and a recent study found an increased cancer risk in males for rectosigmoid- and non-melanoma skin cancers [19, 20].

A missense variant in *MAX* (NM_002382.5; c.220 A > G; p.(Met74Val)), reported as a VUS in ClinVar, was identified in a male (UPN078) who presented with a pituitary gland cancer at the age of 31 years, a pheochromocytoma at the age of 56 years and he also had acromegaly (Table 1). *MAX* is involved in cell proliferation and differentiation and germline PVs in *MAX* have been reported in relation to pheochromocytoma development [21].

We identified compound heterozygous variants in *RECQL4* (NM_004260.4): a VUS (c.3055+5 G > A) that results in loss of a splice donor site and a synonymous variant (c.2082 C > T; p.(Gly694 = )), also classified as a VUS, which potentially creates a novel splice donor site (Supplementary Fig. 3). These variants were identified in a male (UPN114) who presented with macrocephaly, a bladder cancer at the age of 49 years, a colorectal cancer and colorectal polyps at the age of 63 years (Table 1). *RECQL4* encodes for a DNA helicase that is involved in the DNA replication process (OMIM: 603780). Patients with biallelic gPVs in *RECQL4* have been found to develop cancer [22] and other genes that are a part of the *RECQL* gene family, including *RECQL2* (OMIM: 604611) and *RECQL3* (OMIM: 604610) have been associated with syndromes predisposing to both malignant and benign neoplasms [23, 24].

### Likely pathogenic variants in genes involved in cancer-associated pathways

To identify potentially novel genes involved in cancer predisposition, we prioritized variants in genes involved in cancer-associated pathways. For this analysis, we excluded the individuals mentioned above (*n* = 10) and individuals who did not provide consent for analyses beyond the cancer predisposition genes (*n* = 4). We identified a homozygous missense variant in *RECQL5* (NM_004259.7; c.1765 C > T; p.(Arg589Trp)) in a female (UPN083) who presented with short stature (height 154 cm), microcephaly, and a thyroid- and a breast cancer at the age of 46 years. This variant was located in a long region of homozygosity of approximately 12 Mb (Supplementary Fig. 2B). The identified *RECQL5* variant has a nucleotide conservation score of 3.139 (PhyloP) and a CADD_Phred score of 23.9. A prediction tool for missense variant deleteriousness MetaDome, predicts this variant to be in a spot highly intolerant for variation (Supplementary Fig. 4A). This variant is located in a region of *RECQL5* that interacts with *POLR2A* and in silico modeling predicts that the resulting amino acid change affects this interaction (Supplementary Fig. 4B).

## Discussion

In this study, we report the findings of exome-based cancer predisposition gene testing of a retrospective cohort of 72 individuals who developed multiple primary tumors. Overall, in 13.9% (*n* = 10 individuals) of studied individuals, g(L)PVs (*n* = 8 individuals) or VUS (*n* = 2 individuals) in a gene associated with cancer predisposition were identified. These findings are in line with the results of Whitworth et al. who performed WGS in combination with cancer-predisposition gene testing in adults who presented with multiple primary tumors and identified a g(L)PV in 15% of individuals [25]. In 9.7% (*n* = 7) of individuals we identified a g(L)PV that could be associated with the tumor phenotype of the individual and we identified candidate variants in 4.2% (*n* = 3) of individuals. Furthermore, by prioritizing variants with pathogenic potential in genes involved in cancer-associated pathways, we identified *RECQL5* as a candidate gene for further follow-up.

We identified two variants (2 individuals) that could have been, based on the phenotype of the individual and previous gene testing identified, expected. The female with the *NF1* gPV had a clinical diagnosis of neurofibromatosis approximately two decades ago, but was not genetically tested for *NF1*. Based on her development of a thyroid cancer and multiple breast (pre-)malignancies, she was sequenced in the research setting. It is now known that *NF1* gPVs may increase risk to develop breast cancer and there have been reports of thyroid cancer [26, 27]. Therefore, we consider this *NF1* gPV to fully explain the phenotype. The individual with the splice site variant in *PTEN* was previously tested for *PTEN*, but the variant was at that time reported as a VUS. Currently, other variants are reported at this same location to cause alternative splicing and our mini-gene data supports alternative splicing and as such we consider this variant to be causative. The phenotype of the individuals with biallelic gPVs in *CHEK2* (3 individuals) and *FANCM* (1 individual), and the gPV in *POT1* (1 individual), did not completely cover the -at that time- known phenotypic presentation of these syndromes. The extensive phenotypic presentation of these cases is the likely explanation why these cases were not tested for these genes in a targeted manner. In addition to the identification of variants that were almost fully associated with the tumor phenotypes of the individual, we also identified germline variants in cancer predisposition genes that we stated as candidate variants. These candidate variants do not fully explain the observed tumor phenotype or their pathogenicity needs to be determined. For instance, the variant identified in *HOXB13* is associated with the development of the prostate cancer in this individual, but currently no association with melanoma or lung cancer and *HOXB13* germline variants exist. The VUS identified in *MAX* has previously also been identified in a male who developed a pheochromocytoma and a pituitary adenoma [21]. While in an in vitro assay this *MAX* VUS was to some extend unable to repress MYC’s E-box binding ability [28], further supporting functional evidence is needed to determine its pathogenicity. The compound heterozygous variants in *RECQL4* are predicted to have a splice-altering effect, but pathogenicity needs to be confirmed in vitro. Furthermore, the phenotype of the individual is less severe than expected.

In addition, beyond findings in the known cancer-predisposition genes, we identified a homozygous *RECQL5* variant. *RECQL5* is involved in maintaining genome stability as it encodes for a DNA helicase [29] and it belongs to the *RECQL* gene family described above. The region of RECQL5 where the identified variant is located interacts with RNA Polymerase II Subunit A (*POLR2A*) during transcription and suppresses transcription-associated genomic instability [30]. Although computational tools indicate pathogenicity of this variant, more functional work is needed to validate its pathogenicity. However, this finding demonstrates that the investigation of genes other than those already associated with cancer development remains of interest. Novel recessive disease genes or variants are yet to be discovered, which warrants further investigation, for example by applying WGS for a complete picture of all genomic variation [31].

Our study has a few limitations. First, we did not systematically analyze how many individuals with multiple primary tumors obtained a genetic diagnosis from a limited panel of genes. Hence, our study does not reflect to the total population of individuals who develop multiple primary tumors who received genetic counselling. The primary aim of our retrospective study was to describe the series of individuals with heterogeneous tumor phenotypes that obtained a (candidate) genetic diagnoses through WES and not directly evaluate the utility of WES over a targeted gene panel. Therefore, we cannot estimate the overall percentage of individuals with multiple primary tumors who would benefit from the WES approach described in this study. However, as the costs for sequencing are currently still reducing, WES in combination with gene panel-based analysis may become more favorable over targeted gene panel testing. While this approach may allow to identify more VUS and result in unclear diagnosis, it may allow to identify causative variants in genes beyond restricted gene panel testing. Furthermore, this approach may allow for re-analysis of data to yield future diagnosis when novel genes are identified or tools for variant detection in short read-sequencing data become available [32–34]. Second, the cases analyzed in this study were referred for genetic counseling and thus the cohort may be enriched for patients with a suspected family history and/or specific cancer type. For example, a relatively high number of cases in the studied cohort(s) developed breast cancer. However, breast cancer is one of the most common cancers in general, so potentially some cases are phenocopies. Furthermore, other studies also show that among cases with negative large gene panel testing, breast cancers are common [35]. Third, WES data limits the investigation of the non-coding regions of the well-known cancer predisposition genes and investigation of inversions and translocations, which can harbor the possible cause in a proportion of these individuals [36, 37]. Fourth, the exomes analyzed in this study were generated using two different enrichment kits and over various sequencing runs, which hampered copy-number alteration analysis. An alternative to WES that may overcome some of these limitations is WGS. However, the price of WGS and infrastructures required to analyze and interpret the data is still too high in many countries to be implemented in a standard diagnostic setting [38].

In conclusion, we showed that exome-based cancer predisposition gene testing, analyzing all known cancer-predisposition genes, may identify clinically relevant germline (likely) pathogenic variants in individuals who present with heterogeneous (multiple) tumor phenotypes. WES-based cancer predisposition may aid in establishing a genetic diagnosis in individuals with multiple primary tumors, as uncommon diagnoses can be made in individuals who present with non-canonical tumor phenotypes. Such genetic diagnoses have relevant clinical implications, because there are consequences for surveillance regimens to prevent other cancers in the affected individuals and their family members.

## Supplementary information

Supplementary Information

Supplementary Table 1: Overview of the phenotypic information of individuals who developed multiple primary tumors

Supplementary Table 2: Cancer-predisposing genes tested in this study

Supplementary Table 3: Genes from cancer-associated pathways used to prioritize variants beyond known cancer-predisposition genes

## Data Availability

Detailed clinical information is available in Supplementary Table 1.
