# Supplementary material for: Exome-based cancer predisposition gene testing can provide a genetic diagnosis for individuals with heterogeneous tumor phenotypes
Source: Eur J Hum Genet. 2025 Feb 20;33(6):803–9. doi: 10.1038/s41431-025-01814-z (PMC12185683; doi:10.1038/s41431-025-01814-z)
Supplement: Supplementary file 1 — Supplementary Information [file 41431_2025_1814_MOESM1_ESM.pdf]

Supplementary Information to:

Exome-based cancer predisposition gene testing can provide a genetic diagnosis for individuals with heterogeneous tumor phenotypes

Snežana Hinić[1], Arjen R. Mensenkamp[1], Janneke H. M. Schuurs-Hoeijmakers[1], Fulvia Brugnoletti[1,2], Lilian Vreede[1], Elke M. van Veen[1], Barend Mijzen[1], Rachel S. Van der Post[3,4], Maurizio Genuardi[2,3,5], Marjolijn J.L. Ligtenberg[1,3,4], Nicoline Hoogerbrugge[1,3], Richarda M. de Voer[1,3]

Affiliations:

[1]Department of Human Genetics, Research Institute for Medical Innovation, Radboud university medical center, Nijmegen, Netherlands

[2]Genomic Medicine, Department of Life Sciences and Public Health, Università Cattolica del Sacro Cuore, Rome, Italy

[3]European Reference Network for Genetic Tumour Risk Syndromes (ERN GENTURIS)

[4]Department of Pathology, Research Institute for Medical Innovation, Radboud university medical center, Nijmegen, Netherlands

[5]Medical Genetics Unit, Fondazione Policlinico Universitario A. Gemelli IRCCS, Rome, Italy

Supplementary Tables

Supplementary Table 1: Overview of the phenotypic information of individuals who developed multiple primary tumors

see excel file

Supplementary Table 2: Cancer-predisposing genes tested in this study

see excel file

Supplementary Table 3: Genes from cancer-associated pathways used to prioritize variants beyond known cancer-predisposition genes

see excel file

Supplementary Figures

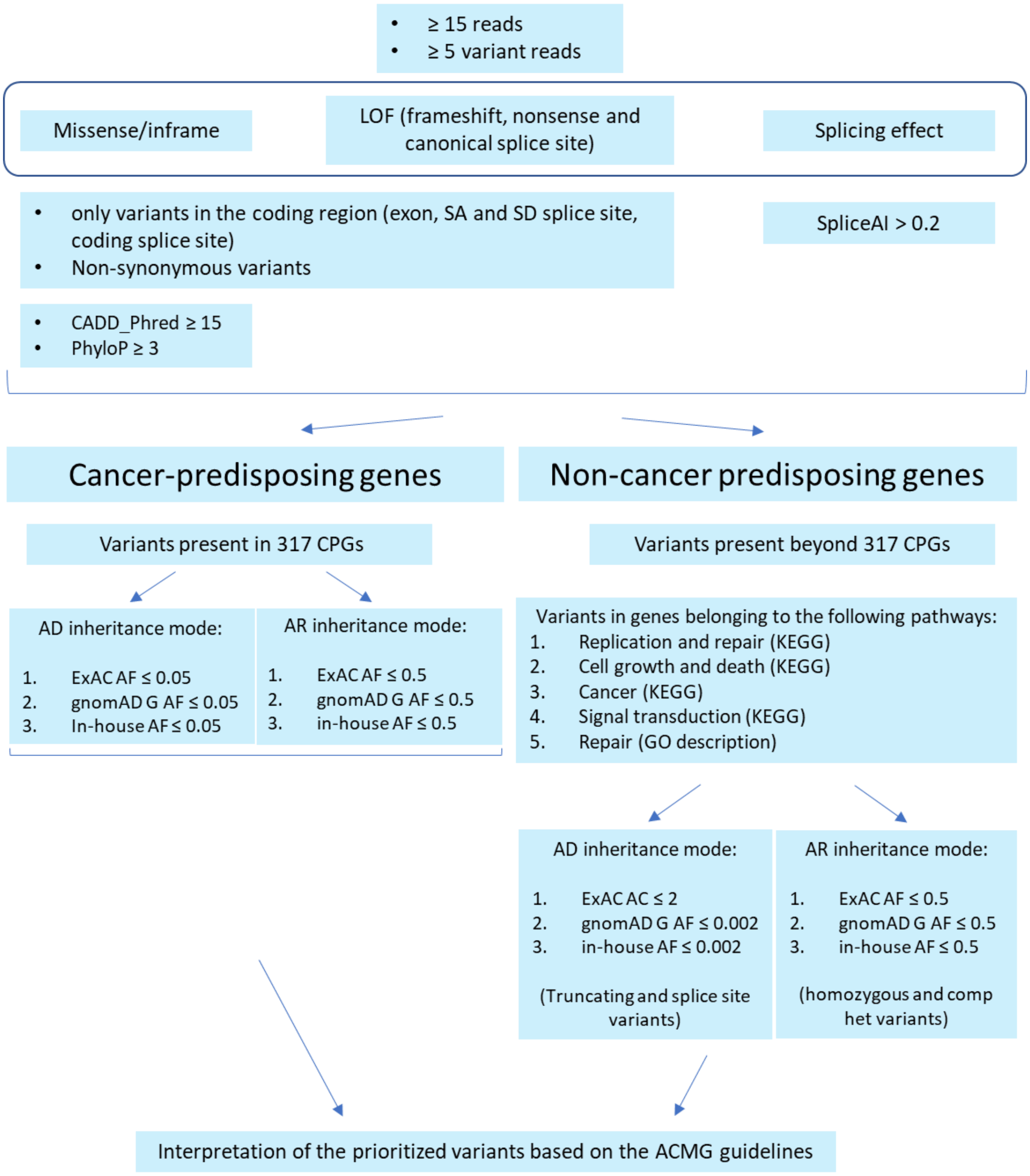

Supplementary Figure 1: Filtering steps used to prioritize germline pathogenic variants in cancer predisposing genes and non-cancer predisposing genes. Abbreviations: AC, allele count; AF, allele frequency; G, genomes; gnomAD, The Genome Aggregation Database; ExAC, The Exome Aggregation Consortium; CPGc, cancer predisposing genes; SA, splice acceptor; SD, splice donor; KEGG, Kyoto Encyclopedia of Genes and Genomes; GO, gene ontology; ACMG, The American College of Medical Genetics; CADD_Phred, Combined Annotation Dependent Depletion; PhyloP, measure of evolutionary conservation; LOF, loss of function.

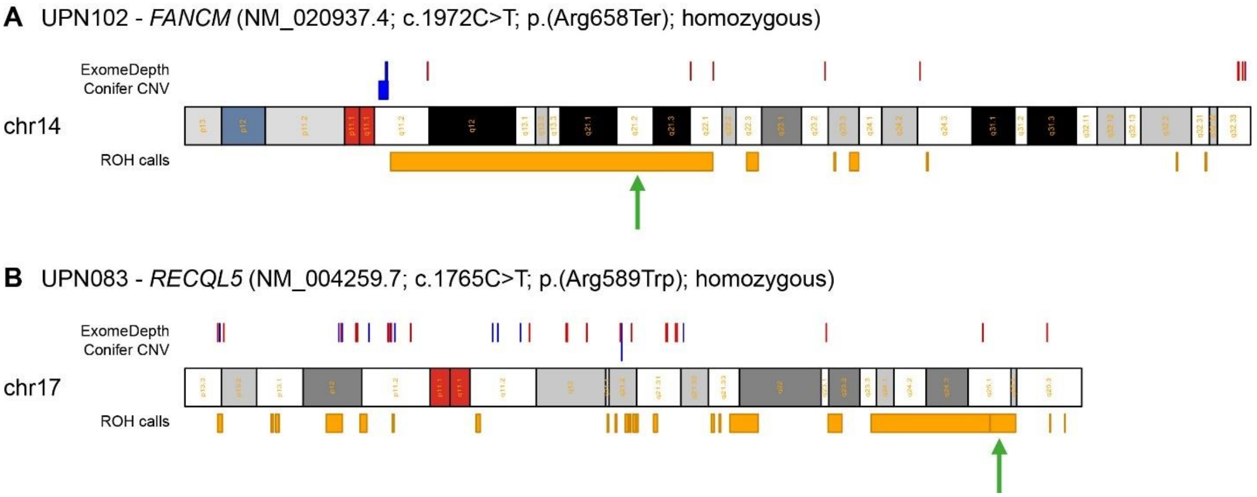

Supplementary Figure 2: Overview of copy number variants (CNV) and regions of homozygosity (ROH). Shown are the chromosome 14 ideogram for **A)** UPN102 and chromosome 17 ideogram for **B)** UPN083. Whole-exome sequencing identified homozygous variants in *FANCM* and *RECQL5*, respectively. Both ExomeDepth and Conifer CNV calls are presented (blue are gains; red are losses). Yellow block represent the regions of homozygous single nucleotide polymorphism (SNP) calls on the chromosome. Green arrows represent the location of the genes of interest. No CNV calls overlap with the aforementioned genes.

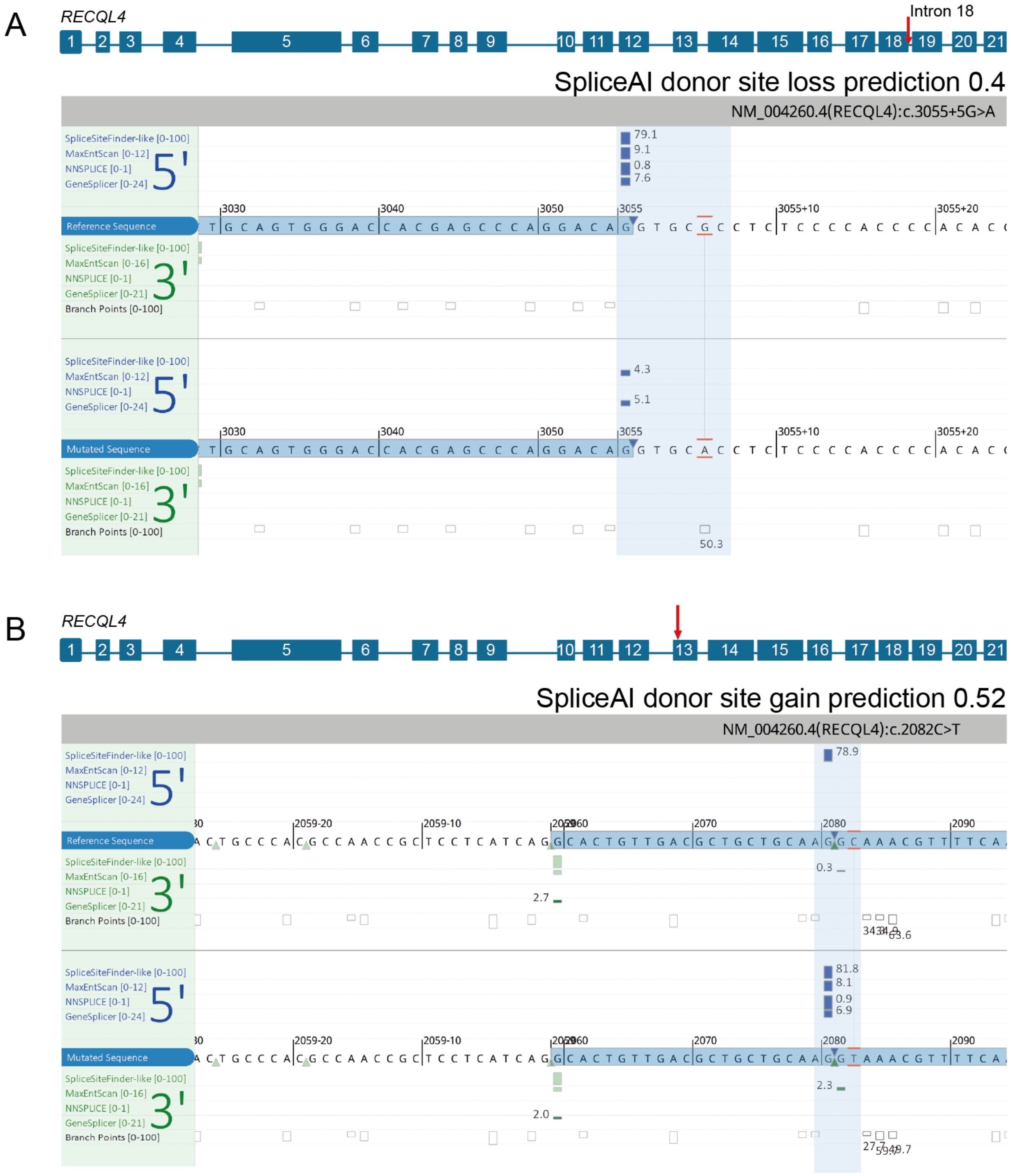

Supplementary Figure 3: Schematic representation of the variants affecting splice sites in *RECQL4* (NM_004260.4). A) Variant c.3055+5G>A is located in intron 18 of *RECQL4*. **B)** Variant c.2082C>T is located in exon 13 of the *RECQL4*. Both variants are labeled with red arrows. For both panels the predicted splicing effects as shown in AlamutVisual Plus are presented. The putative effects on the splice sites are highlighted by the blue squares and the relevant predicted SpliceAI scores are also presented.

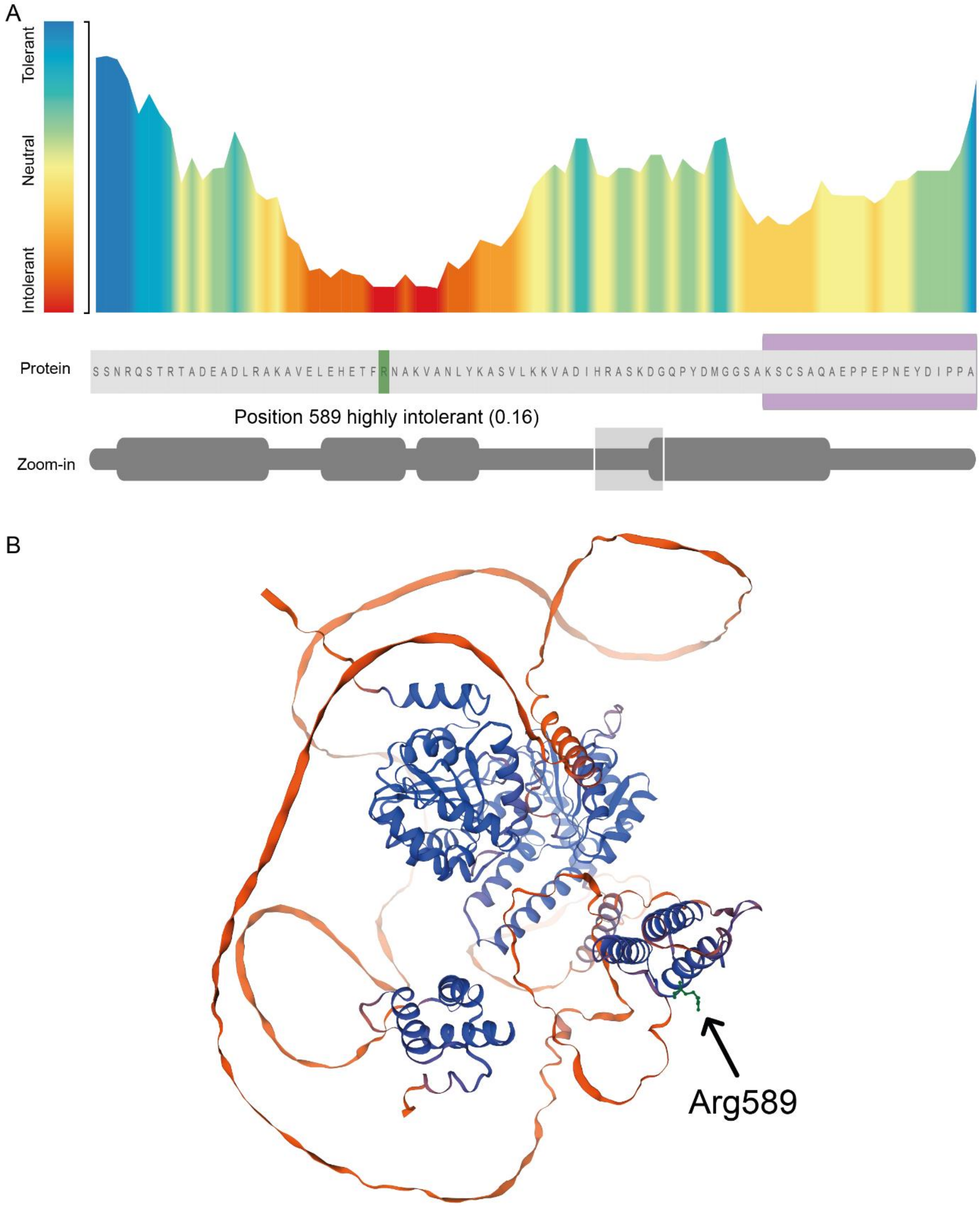

Supplementary Figure 4: *RECQL5* missense variant effect prediction. A) Schematic representation of the RECQL5 intolerance landscape and the location of the identified missense change RECQL5 p.(Arg589Trp), which is labeled in green. Resource: MetaDome web server. **B)** The predicted structure of RECQL5 by AlphaFold. Colors represent the confidence of predicted structure by AlphaFold (gradient between orange and blue representing low to high structure confidence). RECQL5 p.(Arg589) is colored in green. Resource: SWISS-MODEL server.
